# New insight into the role of the pathway NLRP1 and NLRP3 inflammasomes and IL-33 in ultraviolet-induced cutaneous carcinogenesis

**DOI:** 10.3389/fmed.2024.1483208

**Published:** 2025-01-07

**Authors:** Aleksandra Lesiak, Karolina Wodz, Justyna Ceryn, Dorota Sobolewska-Sztychny, Igor Bednarski, Janusz Piekarski, Marta Pabianek, Dariusz Nejc, Izabela Dróżdż, Joanna Narbutt, Marcin Noweta, Magdalena Ciążyńska

**Affiliations:** ^1^Department of Dermatology, Paediatric Dermatology and Oncology, Medical University of Łódź, Łódź, Poland; ^2^Laboratory of Autoinflammatory, Genetic and Rare Skin Disorders Medical University of Łódź, Łódź, Poland; ^3^Laboratory of Molecular Biology, Vet-Lab Brudzew, Brudzew, Poland; ^4^Department of Neurology, Medical University of Lodz, Łódź, Poland; ^5^Department of Surgical Oncology, Central Teaching Hospital of the Medical University of Lodz, Łódź, Poland; ^6^Chemotherapy Sub-Department and One-Day Chemotherapy Department, Specialist Oncological Hospital NU-MED sp. z o. o., Tomaszów Mazowiecki, Poland; ^7^Department of Clinical Genetics, Medical University of Lodz, Łódź, Poland

**Keywords:** inflammasome, NLRP1, NLRP3, IL-33, cutaneous carcinogenesis

## Abstract

**Introduction:**

Inflammasomes NLRP1 (NLR family pyrin domain containing 1) and NLRP3 are pivotal regulators of the innate immune response, activated by a spectrum of endogenous and exogenous stressors, including ultraviolet radiation (UVR). The precise molecular mechanisms underlying the activation of these inflammasomes remain unclear. Furthermore, the involvement of interleukin-33 (IL-33) in UVR-induced skin carcinogenesis is not well defined.

**Purpose:**

The objective of this study is to evaluate the expression of interleukin genes (IL-33, IL-18, IL-1β) following the activation and silencing of NLRP1 and NLRP3 at various wavelengths and doses of UV radiation, and to correlate these expressions with pertinent tumor markers (e.g., Gli1, Gli2, FOXO3A, SerpinA1, SerpinA3, and EphB2).

**Methods and materials:**

Cultures of keratinocyte cell lines were exposed to varying doses of UV radiation using specific lamps. To inhibit the expression of NLRP1 and NLRP3 genes, cells were transfected with targeted siRNAs. Gene expression of inflammasome components and effector proteins was quantified using Real-time PCR and ELISA.

**Results:**

There was a marked upregulation in the expression levels of cytokine genes IL-18, IL-1β, and IL-33 upon exposure to UVB and UVA radiation, compared to non-irradiated keratinocytes. Silencing NLRP1 or NLRP3 via RNA interference in primary human keratinocytes resulted in a significant reduction of cytokine gene expression. Additionally, a notable increase in tumor marker gene expression was observed in cells with functional NLRP1 and NLRP3 following UV radiation, whereas silencing these inflammasome genes altered the expression profiles of these markers.

**Conclusion:**

This study provides a pioneering comprehensive assessment of the roles of NLRP1, NLRP3, and IL-33 in the pathogenesis of UV-induced cutaneous carcinogenesis. Our findings substantiate the role of IL-33 as a critical early danger signal elicited in response to inflammatory UV radiation, presumably regulated by inflammasomes.

## 1 Introduction

The cellular and molecular mechanisms by which ultraviolet radiation (UVR) modulates inflammation and immunity is complex and still not completely understood, however, this property of UV is undoubtedly a key event in skin carcinogenesis (1). UVR is a potent complete carcinogen since it acts as both an initiator and promoter of cancer by inducing DNA damage, production of oxidized lipids and the release of immune-modulating cytokines (2). UVR exposure stimulates keratinocytes to secrete abundant pro-inflammatory IL-1-family proteins, IL-1a, IL-1b, IL-18, and IL-33 (3, 4). In normal human skin, keratinocytes constitutively produce only low levels of inactive precursors pro-IL-1b and pro-IL-18, which accumulate in the cell cytoplasm without active secretion. This inactive precursor forms of IL-1b and IL-18 require presence of caspase 1-mediated proteolysis for their maturation and secretion. However, caspase-1 activation is not constitutive, but depends on the UV-induced formation of an active inflammasome complex.

Inflammasomes are cytosolic multiprotein complexes that plays pivotal role essential for a rapid response of the innate immune system to the presence of pathogen-associated molecular pattern (PAMPs), derived from invading pathogens, and damage-associated molecular pattern (DAMPs) induced because of endogenous stress such as ultraviolet radiation (UVR) induces damage (1).

The NLRP3 (NOD-like receptor pyrin domain-containing protein 3) and NLRP1 (NOD-like receptor pyrin domain-containing protein 1) inflammasomes are a multiprotein complex that plays a pivotal role in regulating the innate immune system and inflammatory signaling. Their activation can lead to the production of pro-inflammatory cytokines such as IL-1β and IL-18, which are implicated in various inflammatory conditions and diseases, including cancer. Inflammasome activation by PAMPs and DAMPs resulting in activation of the protease caspase-1 and secretion of the pro-inflammatory cytokines interleukin (IL)-1 and IL-18. Interestingly IL-33, does not require direct caspase-1 processing for its activation, but depends on inflammasome-associated unconventional mechanisms for its secretion (2). There is some evidence to suggest that inflammasome activation, particularly NLRP3, might play a role in skin carcinogenesis. Some studies have shown that NLRP3 inflammasome activation promotes the progression of squamous cell carcinoma (SCC) by fostering a pro-inflammatory environment. Inhibiting NLRP3 can reduce inflammation and potentially lower the risk of SCC development. The role of NLRP1 in skin cancers is less well-defined. However, given its role in inflammasome activation, it is plausible that silencing NLRP1 might also reduce inflammation and thereby the risk of skin cancer. What is more, in the skin, NLRP1 is highly expressed in keratinocytes, the primary cell type involved in other skin cancer – basal cell carcinoma (BCC) (3–5).

Interleukin-33 (IL-33), a member of the IL-1 family of proteins, is regarded as an alarmin released upon tissue stress or damage. The role of IL-33 has been studied in many types of cancer (6). IL-33 acts as a potent modulator of the tumor microenvironment, and the authors of the reports now hypothesize that it has either pro- or anti-cancer effects. IL-33 has been demonstrated to be a promising biomarker for tumor diagnosis, prognosis prediction, and therapy response in several cancer types. Recently, it was shown that IL-33 is associated with a poor prognosis in several cancer types, even though in certain circumstances it acts as a tumor suppressor by triggering an immune response (6, 7).

It is generally established that chronic inflammation can promote tumor growth, but it is unclear how this immunological milieu is created. So far, the influence of inflammasomes on IL-33 expression and its ability to initiate cutaneous tumorigenesis under the influence of UV radiation is unknown. Therefore, it is necessary to explore different doses of the UVA and UVB as a factor creating a tumor-promoting immune environment in the first place to identify targets for cancer treatment and prevention in chronic inflammation.

## 2 Materials and methods

### 2.1 Cell culture and irradiation of cells with UVA and UVB

Human primary BCC/SCC remnant tumor samples were obtained from patients visiting Clinic of Dermatology, Pediatric and Oncologic Dermatology and Department of Oncological Surgery, Medical University of Lodz, Poland. Acquisition of tumor samples and all studies were approved by the local bioethics committee. Tumor diagnosis was based on clinical and histopathological criteria by a dermatopathologist. In this study, the BCC/SCC samples were taken from sun-exposed (forearm, face and neck) and non-exposed (trunk, legs, upperarm) skin sites.

Primary epidermal keratinocytes (PEK) were grown in Dermal Cell Basal Medium supplemented with keratinocyte growth components (Bovine Pituitary Extract – BPE, rh TGF-*α*, L-glutamine, hydrocortisone hemisuccinate, rh insulin, epinephrine, apo-transferrin). A431 (human squamous carcinoma) was grown in EMEM (EBSS) supplemented with 2mM Glutamine, 1% Non-Essential Amino Acids (NEAA) and 10% foetal bovine serum (FBS). TE 354.T cells were growth in Dulbecco’s Modified Eagle’s Medium 10% foetal bovine serum (FBS). All cell lines PEK, TE 354.T, and 431, was purchased from the American Type Culture Collection and cultured according to the manufacturer’s instructions. Cells were seeded at a density of 3×10^3^ cells/cm^2^ into 75 cm^2^ cell culture flasks and cultured at 37°C in a 5% CO2 incubator. Third passage cells were used for the experiments.

For irradiation experiments, keratinocytes were seeded in 24-well plates at a density of 6×10^4^ cells per well. Irradiation was performed on 70% confluent cells. Before irradiation, the medium was removed, and cells rinsed twice with prewarmed PBS. During the UVA irradiation cells were covered with 1 mL PBS, whereas no PBS was added onto cells for UVB irradiation (short periods needed for UVB). For irradiations cells were kept cold on ice to avoid excessive warming of cells. The subconfluent cells were irradiated with a dose of 1.50, 2.00, 5.00, 10.00, 15.00, 20.00 J/cm^2^ of UVA and 0.005, 0.01, 0.015, 0.02, 0.03, 0.04 J/cm^2^ of UVB using a UV lamp (Philips, Eindhoven, Netherlands). Cells were collected 48 h after irradiation. Non-irradiated control cells were subjected to the same procedure except for lack of irradiation.

### 2.2 siRNA transfection

Small interfering RNAs (siRNAs) against NLRP1 and NLRP3 were purchased from Qiagen (Valencia, CA). For siRNA-mediated knock down experiments, the cells were seeded in 24-well plates at a density of 6×10^4^ cells per well. The day after seeding, cells were transfected with NLRP1 and/or NLRP3 siRNA oligonucleotides or negative control (Qiagen Valencia, CA) or positive control siRNA (Qiagen Valencia, CA) using Transfection Reagent in medium overnight. After siRNA interfering cells were irradiated under the same conditions as cells without siRNAs transfection.

### 2.3 Quantitative real-time-PCR

After each treatment, total RNA was isolated from cell lines and tumor samples with using an AllPrep® DNA/RNA/Protein Mini Kit (Qiagen Valencia, CA), according to the manufacturer’s protocol. The concentration of total RNA was determined by measuring the absorbance at 260 nm using a Nano spectrophotometer. Purity of isolated RNA was determined by the ratio of absorbance 260 nm/280 nm >1.9. cDNA was synthesized from RNA using High Capacity cDNA Reverse Transcritpion Kit (ThermoFisher, USA) according to the manufacturer’s instructions.

Quantitative real-time PCR was performed using Taq Man probes (ThermoFisher, USA): IL-1α, IL-1β, IL-6, IL-17, IL-18, IL-33, TNFα, caspase 1,2,3,5,7,9, IL-1R, HMGB-1, HSP90, FOXM1, Gli1, Gli2, FOXO3A, serpin 1, serpin 3 and EphB2 and NLRP1, NLRP3 on LightCycler480 system (Roche, USA).

The expression levels of genes were normalized to the expression level of the ACTB mRNA in each sample. For mRNA analysis, the calculation for determining the relative level of gene expression was made using the cycle threshold (Ct) method. The mean Ct values from duplicate measurements were used to calculate the expression of the target gene with normalization to a housekeeping gene used as internal control and using the formula 2-ΔΔCT.

### 2.4 Statistical analysis

Results were recorded as mean SD and analyzed using Student’s t test. *p*-values of less than 0.05 were considered significant.

## 3 Results

**Figure 1:**
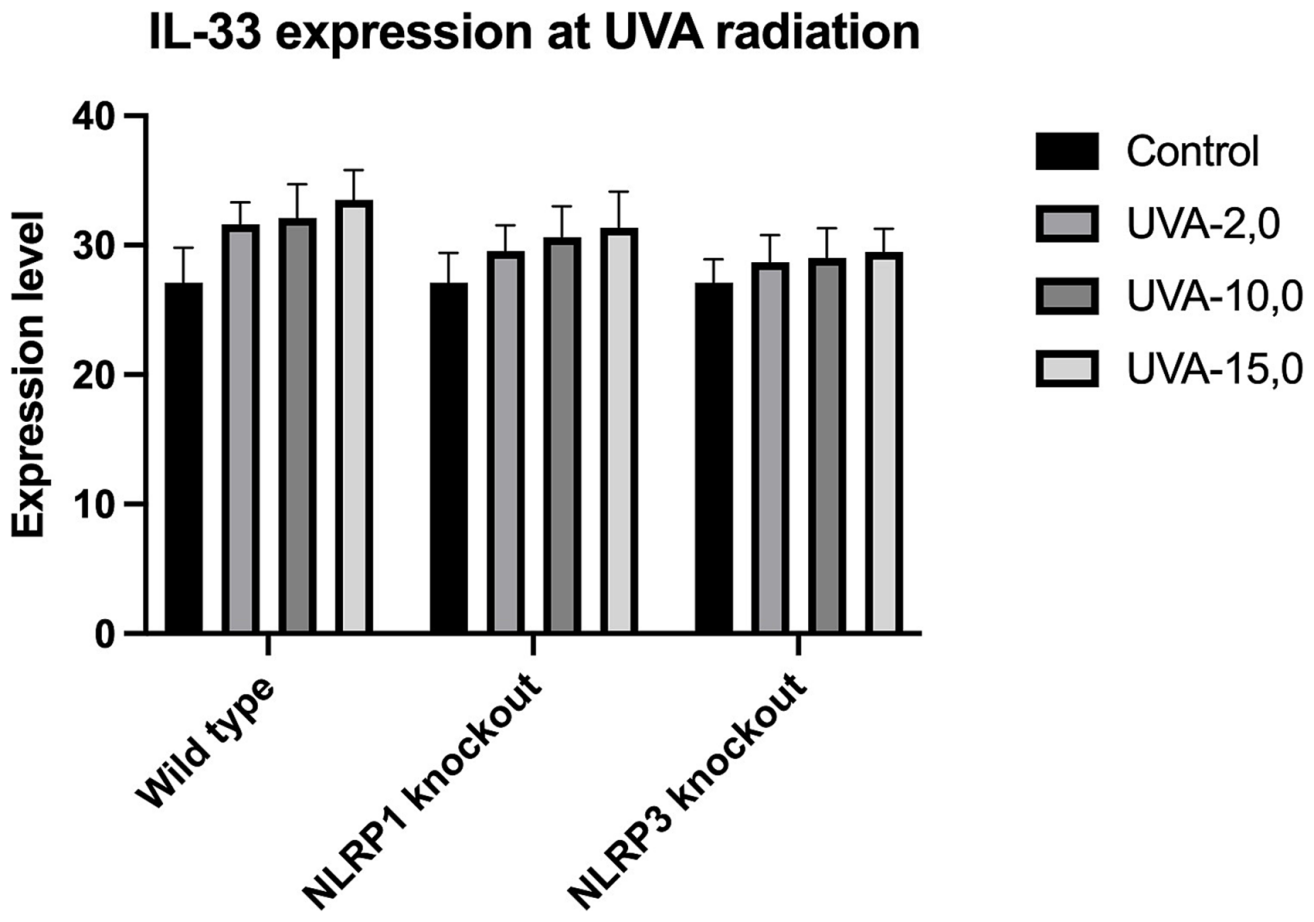
IL-33 expression at UVA radiation.

**Figure 2:**
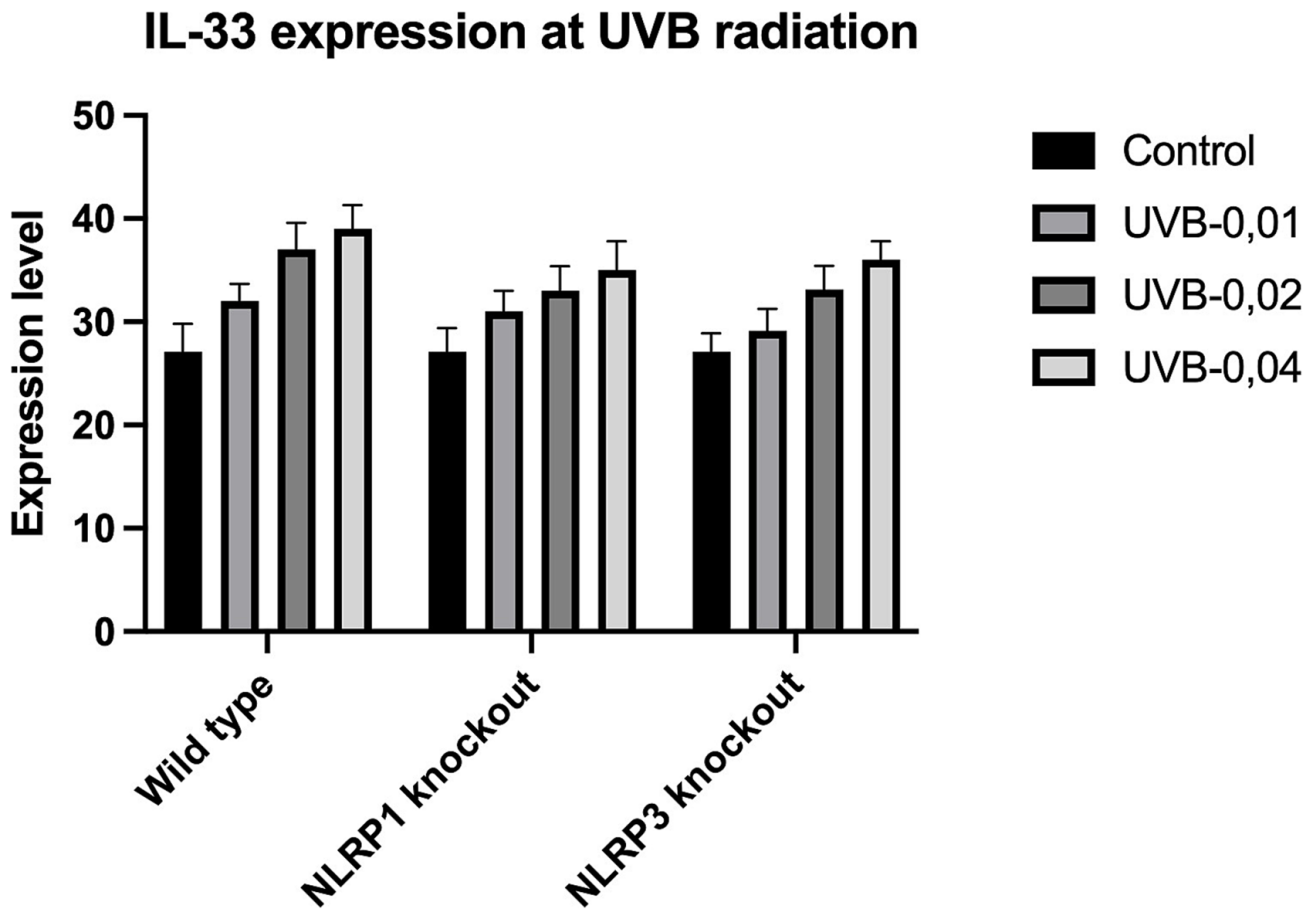
IL-33 expression at UVB radiation.

**Figure 3:**
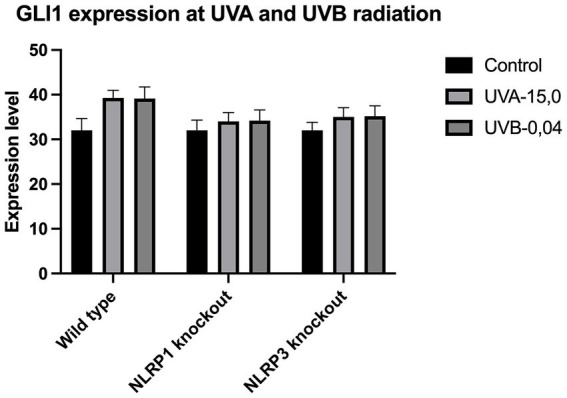
GLI1 expression at UVA and UVB radiation.

**Figure 4:**
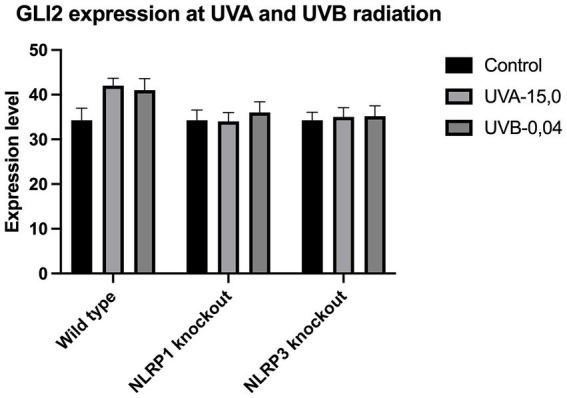
GLI2 expression at UVA and UVB radiation.

**Figure 5:**
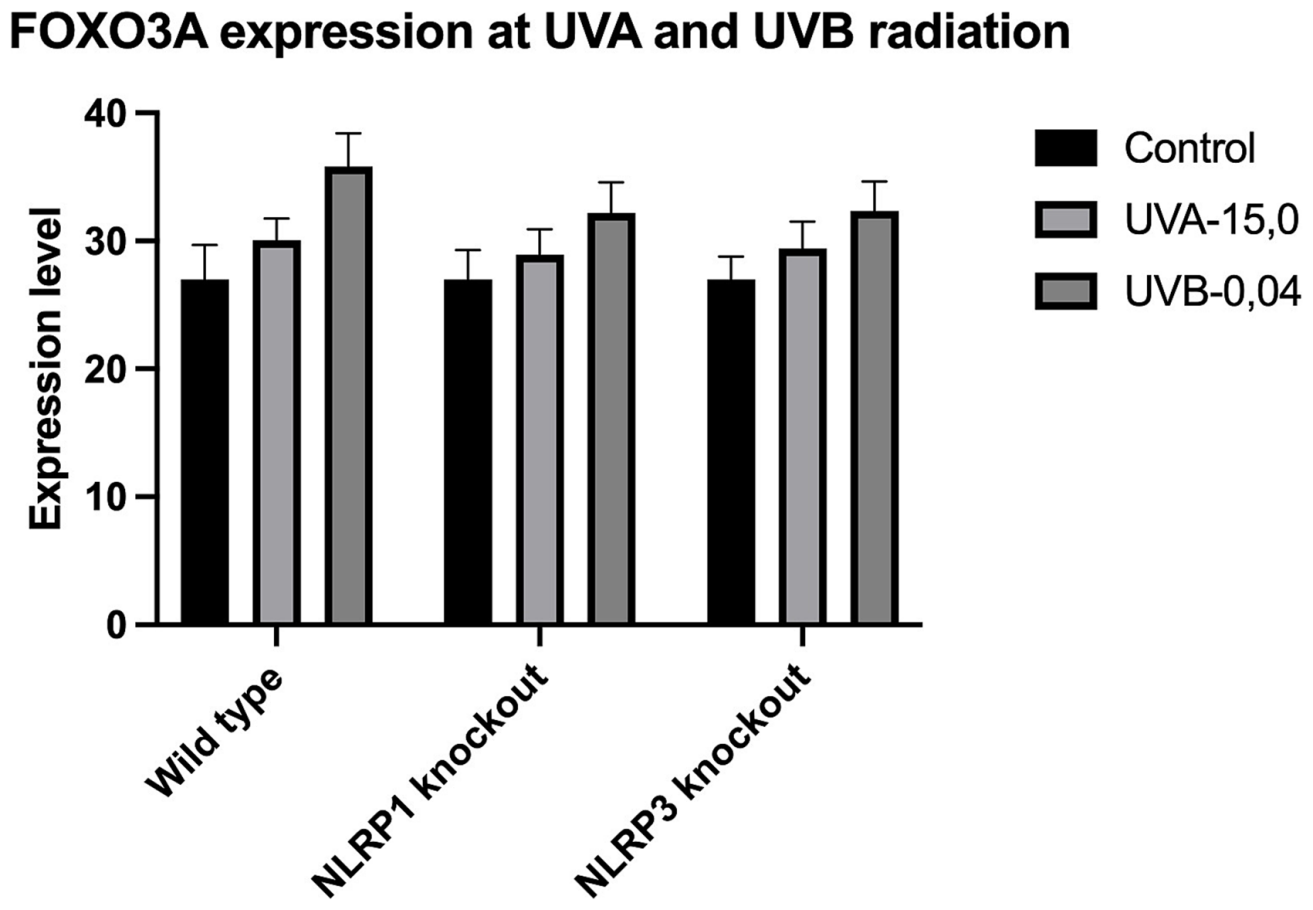
FOXO3A expression at UVA and UVB radiation.

**Figure 6:**
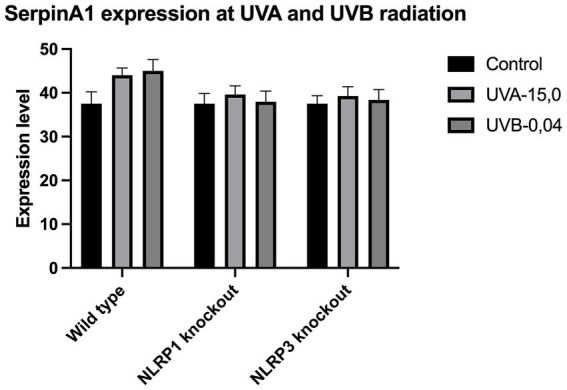
SerpinA1 expression at UVA and UVB radiation.

**Figure 7:**
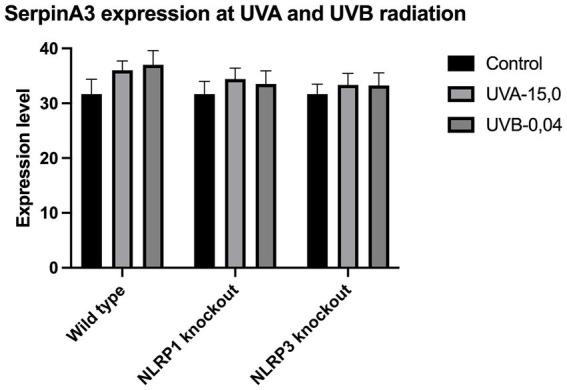
SerpinA3 expression at UVA and UVB radiation.

**Figure 8:**
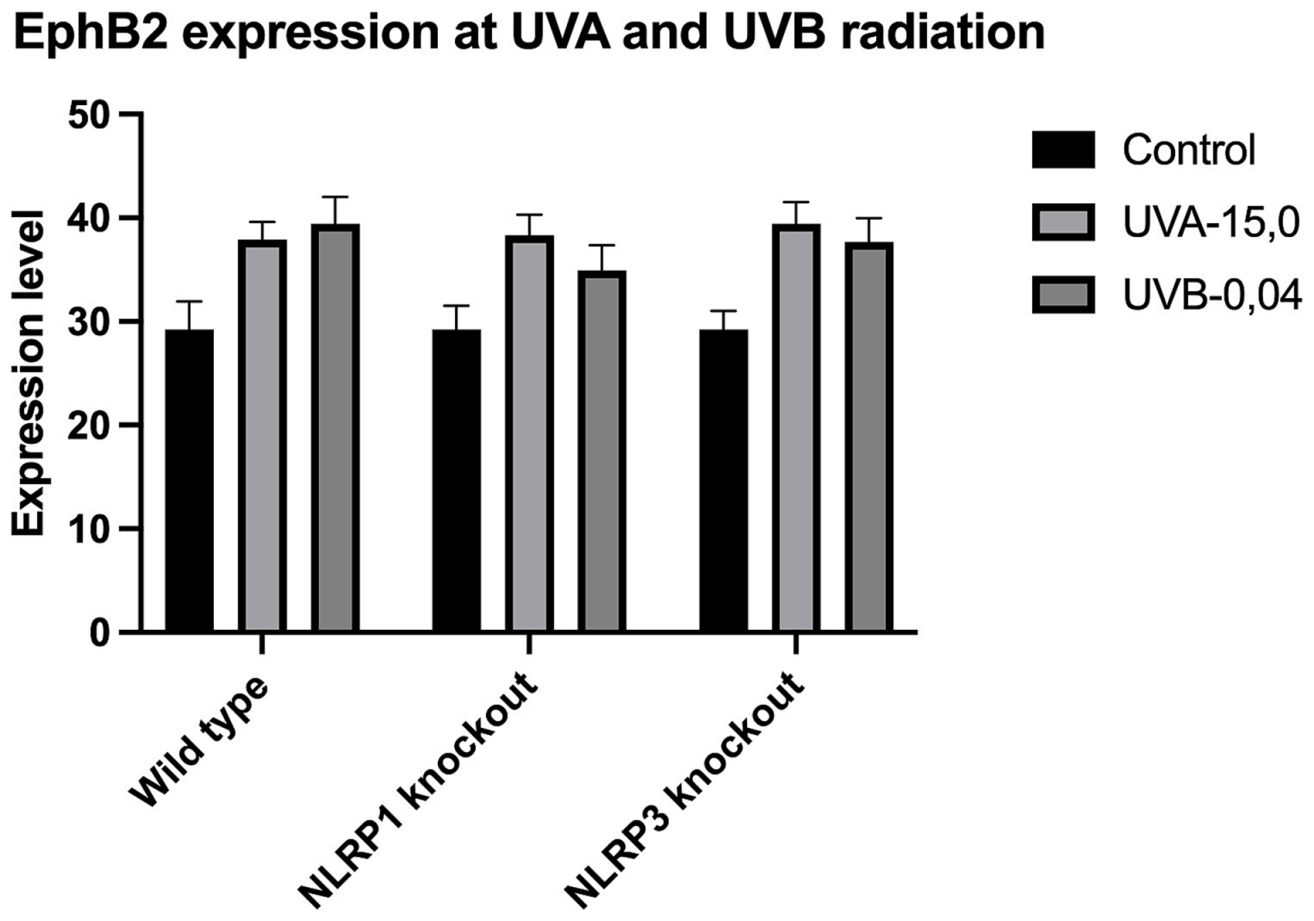
EphB2 expression at UVA and UVB radiation.

The expression of IL-33 increases under the influence of UVA and UVB irradiation. Silencing NLRP1 or NLRP3 reduces the increase in IL-33 expression under the influence of doses of UVA and UVB radiation. Figure 1 presents IL-33 expression at UVA, while Figure 2 presents IL-33 expression at UVB radiation. IL-33 expression was demonstrated in all samples, and its highest value was found in the group irradiated with a dose of UVB-0,04. The median in this group was 39 and was higher compared to the group of unirradiated samples. A slightly lower value of IL-33 expression was observed in the group of samples irradiated with UVA-15,0. The correlation of NLRP1 and NLRP3 silencing and UV radiation with the appropriate BCC tumor markers: Gli1 (Figure 3), Gli2 (Figure 4), FOXO3A (Figure 5) and SCC: SerpinA1 (Figure 6), SerpinA3 (Figure 7), EphB2 (Figure 8) was confirmed. The list of numbers of assay used for the qPCR is presented in Supplementary Table S1. Increased values in irradiated vs. non-irradiated cells clearly indicate that UV radiation induces tumorigenesis.

ELISA analysis of the pro-inflammatory cytokine revealed that both UVA and UVB radiation significantly increased the production of IL-33. Samples were exposed to UVA-1.5, UVA-2.0, UVA-5.0, UVA-10.0, UVA-15.0 and UVA-20.0 radiation, respectively. To determine the role of NLRP3 and NLRP1 in radiation, *Nlrp3^−/−^*, *Nlrp1^−/−^* was isolated from *Nlrp3^−/−^*, and *Nlrp1^−/−^* cultured and then exposed to radiation. Using this strategy, we observed that *Nlrp1^−/−^* and *Nlrp3^−/−^* exhibit resistance to radiation-induced expression of IL-33.

## 4 Discussion

This project is the first comprehensive scientific study to evaluate the roles of the NLRP1 and NLRP3 inflammasomes, as well as IL-33, in UV-induced cutaneous carcinogenesis. Previous studies by Byrne et al. have shown that UVB-induced squamous cell carcinomas, which escape immunological destruction, exhibit significantly elevated levels of IL-33. Our findings are consistent with these observations (8).

While most studies focus on UVB radiation due to its more direct DNA-damaging effects (9, 10), there is evidence suggesting that UVA radiation also contributes to the upregulation of IL-33 in the skin. Our results identify and validate the role of IL-33 as an important early danger signal generated in response to inflammatory UVA and UVB radiation that is presumably regulated by inflammasomes which may have consequences for skin cancer growth.

So far, just two studies have focused on expression of IL-33 caused by UVA. In first study, Meephansan et al. (10) investigated the effect of UVB and UVA on IL-33 expression in cultured normal human epidermal keratinocytes. Their study clearly demonstrated that UVB irradiation of normal human epidermal keratinocytes (NHEK) resulted in a significant increase in IL-33 production through ERK and p38-dependent pathways, but UVA irradiation did not induce IL-33 in NHEKs as no detail data was presented in publication. The lack of impact of UVA was also confirmed by Byrne et al. (8). However, our findings present also elevation of IL-33 caused by UVA radiation and is correlated with doses of UVA radiation, but in smaller impact compared to UVB radiation.

Understanding the cytokine switch will be crucial soon for modulating immune pathways. By controlling the effects of interleukins, we can potentially influence immune responses through various means, including monoclonal antibodies, siRNA, or miRNAs to silence or express key genes. Consequently, our study focuses on the use of siRNA to silence NLRP1 and NLRP3. Elevated NLRP3 levels were shown by He et al., 2018 (11) to be associated with increased downstream inflammatory cytokines, including IL-1β, IL-18, and IL-33, in phosgene-induced acute lung injury. This finding confirms the correlation between NLRP3 and IL-33 expression. However, this relationship may vary depending on the tissue type. Previous research has shown that the NLRP3 inflammasome, along with interleukin-1, may contribute to the growth of skin cancers. In a study conducted on a mouse model, Drexler et al. (12) found that mice lacking interleukin-1 or caspase-1 receptors rarely developed dimethylbenzanthracene (DMBA) and tetradecanoylphorbolacetate (TPA)-induced skin tumors compared to wild-type mice. Additionally, knocking out the NLRP3 gene itself led to a decrease in the number of skin tumors. These findings suggest that NLRP3-dependent production of IL-1β may play a role in the growth of skin cancers. Our results confirm this behavior as silencing NLRP3 could reduce the risk of skin tumors under UVR, as analyzed with skin cancer markers. The superfamily of serine protease inhibitors (serpins) is the largest family of protease inhibitors described in humans. With a size ranging from 350 to 500 amino acids, serpins are considered large molecules compared to other protease inhibitors. Serpins are involved in various biological processes, including inflammation, complement activation, angiogenesis, apoptosis, extracellular matrix maintenance and remodeling, sperm development, and prohormone conversion. Recent research suggests that serpins could serve as prognostic markers for SCC, particularly SerpinA1 (*α*-1-antitrypsin, AAT) and SerpinA3 (α1-antichymotrypsin). It has been found that SerpinA1 and SerpinA3 are upregulated in cutaneous SCC cells compared to normal human keratinocytes. Moreover, SerpinA1 expression correlates with the malignant transformation of epidermal keratinocytes in cell culture and the progression of cutaneous SCC *in vivo* (13). In addition, the EphB2 protein (Erythropietin-producing hepatocellular B2 receptor) appears to be a sensitive and specific marker of SCC progression. Recent studies have shown that EphB2 is a promoter of cell proliferation, thus being a potential therapeutic target for the treatment of SCC (13).

Though undoubtedly the most crucial pathway involved in sporadic BCC development is sonic hedgehog pathway (SHH). Our previous research (14) shown that deregulation of several Shh proteins (Ptch1, Smo, Gli1), leads to sustained proliferation and apoptosis escapement, however not all proteins involved in hedgehog signal transduction could be used as sensitive markers of BCC progression. Gli1 (glioma 1) and Gli2 transcription factors are important downstream effector in the SHH pathway and has been found commonly upregulated in BCC (15). Overexpression of Gli2 also leads to the development of multiple BCC-like tumors in murine model (16). In addition, FOXM1, a Forkhead box protein, is a downstream molecule of SHH that has been found to be overexpressed in BCC and because of its role in cell proliferation it is thought to be one of the causes for aberrant SHH signaling in BCC tumorigenesis (17). Lam et al. (18) found that another Forkhead family protein, the FOXO3A transcription factor, could serve as a sensitive BCC marker due to its role in the negative regulation of cell cycle inhibition, apoptosis, and DNA repair (18).

Our study revealed a significant increase in the level of expression of the cytokine gene IL-33 under the influence of UVB and UVA radiation compared to keratinocytes not exposed to UV radiation. Following RNA disruption by silencing NLRP1 or NLRP3 in human primary keratinocytes, expression of cytokine genes was significantly reduced. Moreover, a significant increase in squamous cell carcinoma proteins marker genes such as SerpinA1, SerpinA3 and EphB2 under the influence of UV radiation was observed in cells with efficiently functioning NLRP1 and NLRP3. On the other hand, silencing the NLRP1 and NLRP3 gene changed the expression of these squamous cell carcinoma proteins marker genes as well as basal cell carcinoma proteins such as Gli1, Gli2, FOXO3A.

We have confirmed the correlation between IL-33 and markers like SerpinA1, SerpinA3, and EphB2 for SCC and Gli1, Gli2, FOXO3A for BCC as it highlights the complex interplay of cytokines, protease inhibitors, and receptor signaling in cancer. These interactions can collectively contribute to tumor growth, immune evasion, and metastasis. Understanding these relationships requires further investigations.

## 5 Limitations

This study had several limitations. Firstly, the number of analyzed cases was limited. Therefore, the study requires repetition on a larger study group and the inclusion of only Polish skin cancer tissue patients may limit the generalizability of the findings from his cancers. Secondly, although we found silencing these inflammasome genes altered the expression profiles of these markers and was closely related to the IL-33 expression, further research into the mechanism involved will be worth pursuing. Thus, first protein expression analysis performed in the ELISA study should be confirmed by the Western blot study.

## 6 Conclusion

This article investigates the roles of the NLRP1 and NLRP3 inflammasomes and interleukin-33 (IL-33) in the initiation of UV-induced cutaneous carcinogenesis. IL-33 is implicated in the onset and progression of skin cancer through its promotion of chronic inflammation, enhancement of tumor cell survival and proliferation, and modulation of the immune response. Our findings highlight IL-33 as a critical early warning signal triggered by inflammatory UV radiation, likely regulated by inflammasomes. Understanding the complex mechanisms by which IL-33 contributes to skin cancer can facilitate the development of novel therapeutic strategies against UV-induced cutaneous carcinogenesis.

Although silencing NLRP1 or NLRP3 could theoretically reduce the risk of squamous cell carcinoma (SCC) or basal cell carcinoma (BCC) by diminishing chronic inflammation and altering the immune microenvironment, further research is essential to comprehensively elucidate their roles and the implications of such interventions. Studies employing animal models and cell cultures are crucial to delineate the full impact of NLRP1 and NLRP3 silencing on SCC and BCC risk. These investigations can clarify the balance between alleviating harmful chronic inflammation and preserving essential immune functions.

Therapies aimed at selectively modulating NLRP1 and NLRP3 activity, rather than complete silencing, may offer a more balanced approach. This could be explored through selective inhibitors or gene-editing technologies, such as CRISPR, which may provide a foundation for further, more detailed research. Understanding these mechanisms could inform the design of innovative immune-modulating treatment regimens. Nonetheless, extensive experimental and clinical research is required to evaluate the efficacy and safety of targeting these inflammasomes in the prevention and treatment of skin cancers.

## Data Availability

The raw data supporting the conclusions of this article will be made available by the authors, without undue reservation.
