# Supplementary material for: New insight into the role of the pathway NLRP1 and NLRP3 inflammasomes and IL-33 in ultraviolet-induced cutaneous carcinogenesis
Source: Front Med (Lausanne). 2025 Jan 7;11:1483208. doi: 10.3389/fmed.2024.1483208 (PMC11747519; doi:10.3389/fmed.2024.1483208)
Supplement: Supplementary file 1 [file Table_1.pdf]

Supplementary Table 1. List of numbers of assay used for the qPCR.

NLRP1 - Hs00248187_m1
NLRP3 - Hs00918082_m1
FOXM1 - Hs01073586_m1
GLI1 - Hs00171790_m1
GLI2 - Hs01119974_m1
EPHB2 - Hs00362096_m1
IL33 - Hs04931857_m1
FOXO3 - Hs00818121_m1
SERPINA1 - Hs00165475_m1
SERPINA3 - Hs00153674_m1
